# The Feasibility and Impact of Delivering a Mind-Body Intervention in a Virtual World

**DOI:** 10.1371/journal.pone.0033843

**Published:** 2012-03-28

**Authors:** Daniel B. Hoch, Alice J. Watson, Deborah A. Linton, Heather E. Bello, Marco Senelly, Mariola T. Milik, Margaret A. Baim, Kamal Jethwani, Gregory L. Fricchione, Herbert Benson, Joseph C. Kvedar

**Affiliations:** 1 The Benson-Henry Institute for Mind Body Medicine, Boston, Massachusetts, United States of America; 2 Center for Connected Health, Partners Healthcare, Boston, Massachusetts, United States of America; 3 Dermatology, Massachusetts General Hospital, Boston, Massachusetts, United States of America; 4 Neurology, Massachusetts General Hospital, Boston, Massachusetts, United States of America; 5 Harvard Medical School, Boston, Massachusetts, United States of America; 6 Harvard School of Public Health, Massachusetts, United States of America; 7 Health 2.0, San Francisco, California, United States of America; 8 OneVision Solutions Inc., Austin, Texas, United States of America; 9 Psychiatry, Massachusetts General Hospital, Boston, Massachusetts, United States of America; Research and Development Corporation, United States of America

## Abstract

**Introduction:**

Mind-body medical approaches may ameliorate chronic disease. Stress reduction is particularly helpful, but face-to-face delivery systems cannot reach all those who might benefit. An online, 3-dimensional virtual world may be able to support the rich interpersonal interactions required of this approach. In this pilot study, we explore the feasibility of translating a face-to-face stress reduction program into an online virtual setting and estimate the effect size of the intervention.

**Methods and Findings:**

Domain experts in virtual world technology joined with mind body practitioners to translate an existing 8 week relaxation response-based resiliency program into an 8-week virtual world-based program in Second Life™ (SL). Twenty-four healthy volunteers with at least one month's experience in SL completed the program. Each subject filled out the Perceived Stress Scale (PSS) and the Symptom Checklist 90- Revised (SCL-90-R) before and after taking part. Participants took part in one of 3 groups of about 10 subjects. The participants found the program to be helpful and enjoyable. Many reported that the virtual environment was an excellent substitute for the preferred face-to-face approach. On quantitative measures, there was a general trend toward decreased perceived stress, (15.7 to 15.0), symptoms of depression, (57.6 to 57.0) and anxiety (56.8 to 54.8). There was a significant decrease of 2.8 points on the SCL-90-R Global Severity Index (p<0.05).

**Conclusions:**

This pilot project showed that it is feasible to deliver a typical mind-body medical intervention through a virtual environment and that it is well received. Moreover, the small reduction in psychological distress suggests further research is warranted. Based on the data collected for this project, a randomized trial with less than 50 subjects would be appropriately powered if perceived stress is the primary outcome.

## Introduction

William Cannon first described the concept of “fight or flight”, also known as the stress response, over 80 years ago [1]. Since then, we've also learned that activation of the stress response can affect the course of disease [2]. A contrasting “relaxation response” (RR) has been described by Benson and colleagues as a physiologic state that is counter to that induced by stress [3]–[5]. The relaxation response is accompanied by reduced oxygen consumption and carbon dioxide production [4], [6], diminished sympathetic nervous system tone [7] and increased exhalation of nitric oxide [8]. Functional studies of the brain have shown decreased EEG fast activity in the frontal areas [9] and increased activation of the hippocampus and other parts of the limbic system that may moderate emotion [10] as well as other structural changes on imaging [11]–[13]. These changes may reflect some of the reasons that regular elicitation of the relaxation response has been reported to alleviate stress-related medical disorders [14]–[16]. Specific disorders impacted include hypertension and cardiac arrhythmias [17], chronic pain [18], insomnia [19], [20], anxiety and depression [21], premenstrual syndrome [22], and infertility [23].

Widespread use of the relaxation response is handicapped by the teaching method. It has traditionally required significant face-to-face interaction with clinician-teachers. These face-to-face teaching sessions, delivered weekly for up to 12 weeks, require time and travel. Those with limited mobility have difficulty taking part. Costs, the clinical model, and concerns about privacy in the group teaching model, provide additional hurdles to participation. Fortunately, the Internet has provided a conduit for reaching out to those with limited mobility who may also wish to maintain a certain degree of anonymity even while taking part in a group encounter. While many health interventions have been presented using the Internet [24]–[27], the exceptional opportunities afforded by online, 3- dimensional or virtual environments have not yet been systematically explored for mind-body interventions.

A virtual environment offers many advantages not available in a typical Internet based application. Individuals create representations of themselves, give that representation unique properties to reflect their emotions or feelings and can interact with others in a rich, shared space. One extremely successful application is that of Second Life™ (SL). The free software allows users to interact in a persistent virtual world represented as a character referred to as an avatar. Stock avatars are predominantly human and come with free “animations” that communicate basic body language such as laughing, waving, falling or flinching. While anonymity is not guaranteed, it is difficult to determine the true identity of a user if they chose not to share it.

People have been quick to employ SL for health related purposes. Self-organizing patient collectives were the first to develop, usually with expert patients at their core. However, the last few years have seen an increase in formal organizational involvement [28]. For many, these communities offer a degree of emotional support that cannot be achieved with existing telehealth solutions. Mainstream health care providers and organizations are also exploring this virtual environment as a tool to deliver technology-enabled care [29].

In this pilot study we aimed to determine if it is feasible to teach people to elicit the relaxation response using SL as the clinical teaching medium and to determine if there is an impact on perceived stress and other psychological symptoms. If successful, this approach could make it possible to reach a wider audience of individuals who could benefit from this mind body approach to health.

## Methods

The project was designed as a pilot study to find out if it is logistically feasible to teach stress reduction in an online virtual world and if so, to determine the effect size. The data could be used to design a larger, randomized trial of the program. It was an open trial with no blinding or randomization. The target for recruitment was 20–40 healthy volunteers. This number of patients was selected based on the experience of clinicians at the Benson-Henry Institute with face-to-face groups where a small aggregate effect was seen in groups of 15–20 participants. We hypothesized that the virtual program would be less effective and adjusted our target to 20–40 subjects divided into 2–3 groups.

The project was reviewed and approved by the Partners Human Research Committee (PHRC) which serves as the Institutional Review Board of Partners Research Management. During the review of the Project, the PHRC specifically considered (i) the risks and anticipated benefits, if any, to subjects; (ii) the selection of subjects; (iii) the procedures for securing and documenting informed consent; (iv) the safety of subjects; and (v) the privacy of subjects and confidentiality of the data. Informed consent was obtained from all participants in the study, in person by a member of the research team. Each participant was given an opportunity to read the consent form at their leisure and to ask questions prior to signing the form.

Patients were recruited by word of mouth in virtual world communities, through advertisement kiosks in the virtual world, through contacting local SL users groups and through a presentation at the Boston Linden Lab office. A local newspaper article in the Boston Globe about the project also raised awareness and was helpful for recruitment.

The inclusion and exclusion criteria are shown in Table 1. The user interface for SL is somewhat difficult to learn and so only individuals with experience in SL were recruited to minimize the confounding effect of the interface. In addition, individuals had to be willing to attend two face-to-face meetings at the Massachusetts General Hospital. This requirement served two important purposes, to obtain standard, in-person, informed consent and to use surveys presently validated only for face-to-face or telephone administration.

**Table 1:** Inclusion and exclusion criteria.

Inclusion Criteria:	Exclusion Criteria:
Over 18 years of age	Engaged in ongoing psychiatric therapy or formal relaxation training
Must have a computer with speakers and internet access in a secure setting (home or private office)	The presence of significant psychotic symptoms
User of the virtual world SL for at least one month prior to entry in the study	
Able to attend two face-to-face meetings in Boston	
Fluency in English (spoken and written)	

### Development of the virtual program

The virtual program was developed by drawing on the expertise of clinicians and team members with experience in SL. A group of 2 mind body trainers (MAB and MTM) and 2 developers (DAL and MS) met bi-weekly with facilitation by the PI and project manager (HEB), to share their knowledge. This division of expertise permitted the clinical team to focus on the conversion of their practice to SL while allowing the technical team to best understand how to best recreate the face-to-face experience in this virtual environment.. Although the face-to-face teaching area is somewhat austere, the virtual space was designed to be comfortable and welcoming, but without too many distractions.

**Figure 1:**
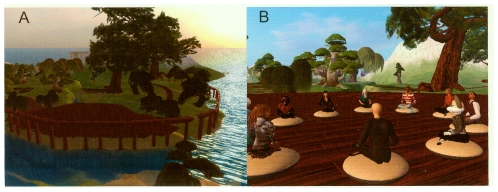
Images of virtual environment. Two views of the virtual teaching area. Panel A shows a part of the forest path, leading to the teaching area. Walking along this path, subject would collect materials for the weekly session and make the transition into the program. Panel B shows the teaching area from above. This area was used for presentations, yoga and meditation.

The team integrated standard web technology into the environment to simplify the development. By using external links to standard web sites for streaming video or online surveys the team could focus on the unique technical opportunities offered by this virtual world. Representative pictures of the virtual environment are shown in Figure 1 while a list of principle features is presented in Table 2.

**Table 2:** Key Features of the virtual environment.

Teleport site at head of a forest boardwalk
Boardwalk, with 8 sites at which to pick up weekly materials to be added to inventory at intervals along the walk
Teaching and meditation platform, screen for presentations
Meditation cushions, yoga mats
Labyrinth for walking meditation in forest area near teaching platform
Wind chimes as backdrop to meditation
Audio listening posts for subjects to use at their leisure as an aide to practice, spread throughout the area.

The participants were given some limited ability to modify the virtual environment. We felt thatif the groups could make the virtual space more personal, it would promote greater group cohesion. Mature content and software that would interfere with or slow the function of the environment were not allowed.

### Procedures

Each subject completed baseline questionnaires including an intake history and pre-training survey as well as psychosocial self-assessment instruments. The latter were chosen because of ongoing experience with the impact of teaching the RR on these measures [30]. They included the SCL-90-R, a general psychological symptom checklist and the Perceived Stress Scale. After this initial face-to-face meeting, each subject was given the date and time of the initial group meeting in SL.. Subjects were assigned to a group in the order that they enrolled. Each of the 3 groups included approximately 10 people. Participants were also given a copy of the consent form, a $20 American Express Check to cover their parking/travel expenses for the face-to-face meeting, and a microphone-headset to facilitate their engagement in the program in SL (value = ∼$100).

### Program Details

The groups were facilitated by the same clinician (MAB). The clinician had over 20 years of experience teaching mind body medical interventions at the BHI. She is a nurse practitioner with a Masters in Nursing and specialty training in cognitive applications of positive psychology and contemplative meditation. Each group gathered in the virtual meeting area with the clinician twice per week for 8 weeks. The content of each of the 8 weeks is given in table 3.

**Table 3:** Program content and supportive materials or exercises by week.

Week	Technique used to elicit the RR	Supportive materials or exercises
1	Imagery	PowerPoint presentation on link between meditation and resiliency
	Desired self and Safe Place	Review of meditation strategies and methods
	Single-pointed focus of breath, word, image or phrase	Examples of mini meditation exercises
	Body scan	Guided imagery scripts
2	Body scan,	Compact Disc: Two meditations on building focus, positive imagery and mindfulness
	Pulse-focused meditations	PBS video featuring 4 applications of the relaxation response
	Mindfulness	Opening the heart meditation script from Sufi tradition
		Review of mindfulness
		Gratitude research review
3	Smile meditation	
	Hatha Yoga Mudras	Avatars programmed for 5 yoga poses-Extensive review of Hatha yoga with diagrams to support postures
4	Emotion-insight guided imagery	Compact Disc: guided emotion-insight meditation
		Emotion-insight imagery script
5	Wise person guided imagery	Compact Disc: guided wise person imagery
		Selected excerpts from *Meditation*s by J. Krishnamurti
		Walking meditation script
6	Contemplation of positive qualities	Compact Disc: guided contemplation and guided imagery focusing on heart's desire
		Review concept of contemplation
		Contemplation script
7	Contemplation of positive qualities	Excerpt from *Simple Truths: Clear & Gentle Guidance on the Big Issues in Life* by Kent Nerburn
8	Tonglen, Guided loving	Compact Disc: guided tonglen
	kindness meditation	Review of tonglen
		Tonglen script

Five different techniques for eliciting the RR were taught: 1) breath awareness, 2) mental repetition of a word, sound, phrase, or prayer, 3) mindfulness meditation, 4) guided body scan and Hatha Yoga, and 5) guided imagery. These 5 techniques are commonly used for eliciting the relaxation response and were used in previous studies of patients and healthy volunteers. The availability of more than one technique allows for individuals to explore several approaches and to avoid overuse of a single technique which may become tedious and a barrier to adherence. Instructional audio files of 20-minutes in length on each of these techniques were provided to subjects through SL for practice throughout the study. The other exercises described in Table 3 are felt to enhance resiliency and are commonly paired with eliciting the RR in mind body programs.

The first meeting of each week lasted approximately an hour and was an overview of the principals of mind body interventions, the nature of the relaxation response, and an opportunity to learn and practice several methods to elicit the relaxation response. The second meeting each week lasted 20 minutes and was offered to answer questions and reinforce the teaching present at that week. Subjects were instructed to elicit the RR every day for 20 minutes either in front of the computer with their avatar (representation of themselves) in the virtual teaching area, or in any other quiet setting. A relaxation audio recording was available at specific locations in the virtual world, activated by guiding the avatar to that area at any time. Participants were not allowed to download the audio file for use off line.

Subjects reported on their engagement in the program weekly using an online survey. The survey asked them to report the number of days they practiced for 20 min or more, methods they used for practice, the number of “mini” breathing relaxation exercises they used daily, and to report on symptoms they may have experienced.

### Statistics

The areas of interest for this pilot study included three scales on the Symptom Checklist 90-Revised (SCL90-r) and the Perceived Stress Scale (PSS). The scales for the SCL90-r that were examined included the Global Severity Index (GSI) which measures overall psychological distress, the depression scale which measured symptoms of depression (DEP) and a scale for anxiety-related symptoms (ANX). Statistical analysis was performed using STATA on these measures that were obtained before and after taking part in the program. Since this was a pilot program in which a small number of individuals took part, we were unable to stratify by potential covariates such as gender, age and educational level.

## Results

A total of 55 individuals were screened to enroll 28 subjects. The only reason for ineligibility was inexperience with SL. A complete data set for analysis was obtained in 24 of the 28 volunteers, because 4 subjects met the objective drop criteria of the study and were not included in the final analysis. (One failed to answer all questions on the pre-program surveys but competed the entire study, two completed the course but incompletely filled out the post-program surveys and one did not attend the final face-to-face meeting). Since the study was designed as a feasibility pilot, and was not powered for statistical significance, we have excluded their data comletely. A description of the 28 participants is given in Table 4.

**Table 4:** Characteristics of the study participants.

Total number of volunteers completing the program, N = 24		
Mean Age	42 y	S.D. = 13
Gender	57%	Male
Race	86%	White
Marital Status	54%	Married
Educational Level	100%	College and above
History of counseling	64%	Any psychotherapy (self report)

### Impact of the intervention

A total of 24 of the 28 subjects completed both pre and post program surveys. Scores on the Perceived Stress Scale and all 3 of the scales of the SCL90-r were normally distributed and compared pairwise. There was a trend toward improvement in each measure. Change on the Global Severity Index of the SCL 90-r reached statistical significance as shown in Table 5.

**Table 5:** Scores on surveys of stress and psychological symptoms.

Survey Instrument	Pre-program (X± S.D)	Post-program (X± S.D)	Change	Statistical significance
PSS	15.7±5.9	15±6.7	−0.7	0.501
SCL-90r:				
−GSI	57.8±7.9	54.9±9.9	−3.9	0.024*
−DEP	57.6±9.4	57.0±10.4	−0.6	0.683
−ANX	56.8±10.2	54.8±11.4	−2.0	0.126

PSS = perceived stress scale, SCL-90r = Symptom Checklist 90 revised, GSI = Global Severity Index of the SCL-90r, DEP = Depression index of the SCL-90r, ANX = Anxiety Index of the SCL-90r.

P-value<0.05.

### Qualitative Results

The individuals taking part in this study were extremely positive about the experience although most did not attend all of the sessions in SL. Two of the best accepted components of the traditional, face-to-face program: guided imagery and single pointed focus, were reported by a majority of our subjects to be “the best aspects and best takeaway from the program”. Thus, there is some consistency in the reaction to the program presented in the virtual world and face-to-face.

Many participants reported that were it not for the convenience of remotely logging in for the one hour session, they would not have been able to commit to the program schedule. In addition, some felt that the subject matter was more approachable and digestible due to the anonymity. Several direct quotes from participants demonstrate this feature well:

*“I would probably not go to a study like this with strangers in the real world… the virtual space felt like a very safe environment….you can control what you reveal about yourself…. There is no way I would have done this if I had needed to participate in class physically, plus think about the travel time.” (participant 407)*

*“I liked the fact that we could sit for hours doing these guided mediations together and stand up and not be stiff - doing this via SL in my comfortable chair made it easier to not focus on bodily aches and pains.” (participant 202)*

## Discussion

The goals of this pilot study were to determine if it is feasible to convert a face-to-face mind body program into one that can be delivered in a virtual setting, and to estimate the effect size in this setting for the design of a larger clinical trial. Both goals were achieved, and important lessons were learned.

Although somewhat challenging, providing the program in a virtual environment is feasible and it would not require an unusually large sample to definitively test the efficacy of the program. The perceived stress scale would be a reasonable choice for primary outcome in such as study. It has excellent face validity and is easy to understand. Additionally there is a wealth of information about the perceived stress scale because “stress” is a common complaint in the general population and in clinical practice and Cohen and his collaborators have recently compared perceived stress in a relatively large sample of individuals [31].

Several important lessons were learned in the course of this study. First, although it is indeed feasible to present this type of program in a virtual world, the user interface is problematic. Recruitment was limited to individuals with prior experience in SL since the interface was known to be a barrier to entry. Even with such inclusion criteria, some of the less experienced users had problems that likely affected their participation. Prescribing this type of technology to individuals at large, with no experience in SL, would only be possible if some type of face-to-face tutorial or training program was also offered.

There are several important limitations to the use of a virtual environment as described in this report. First, the healthy subjects we recruited were relatively young and exceptionally well educated. Since much chronic illness, especially that which limits mobility, is found in the older population, a virtual environment could be very difficult to use without significant technical support. Further, in the absence of remote sensing technology, it is very difficult to know if the participants are taking part in the exercises or succesfully eliciting the relaxation response. However, improvements in access to ease of use of technologies may moderate both these concerns.

Technology changed significantly even in the short span of this study. When we initiated the project, the audio capabilities of SL were markedly unreliable. We decided to use Skype™ for the audio component. However, shortly after our study began, Linden lab improved SL “voice” and it is now adequate for this type of program. There is still an advantage to a separate audio technology, discovered as an unanticipated consequence of our design. If one system fails, the other persists and communication is intact.

A final limitation due to the unique profile of our subjects is that of stress level. In comparison to the normative data recently reported by Cohen [31], our sample reported a perception of greater stress than the general population. This may be a function of their high education that could result in more demanding jobs, local stressors or any number of other reasons that we did not address.

Providing care in a virtual world such as SL also presents a number of theoretical, practice-related challenges. Most malpractice is alleged due to negligence and proving negligence requires four elements: duty of care, breach of duty, injury and proximate cause. Further, one of the tenets of successful provider risk management is clear, caring communication with the patient. In a virtual world such as SL, challenges may arise in the clear establishment of duty, communication of a breach, and in documentation of injury or proximate cause. Likewise, great challenges around communication arise when the vehicle for communication is an avatar and exchange of text messages. Cues that are used to sense the clarity of communication such as eye contact and body language are missing. The anonymity associate with the virtual world can cloud all aspects of the provider patient relationship. The cues that one uses to confirm an individual's identity in a face-to-face meeting are lost. It is easy to masquerade as another individual in the virtual world since most contact occurs via text communication, albeit our use of voice helps. Working in a virtual world would place additional burden on a clinician to be clear about availability (since she will be in the virtual world for limited times) in order to avoid allegations of negligence. It must be emphasized that these concerns are indeed theoretical, as no case law has emerged on what is both a nascent and low-volume activity.

There are good reasons to continue to devise and investigate methods for widely distributing stress management programs like the one presented in this study, in spite of the challenges described above. The American Institute of Stress estimates that from 75 to 90% of primary care visits are to some extent related to stress. Face-to-face mind body interventions have been shown to be beneficial for many stress related conditions. However it will be difficult to make these self care strategies available on the scale necessary for effective primary, secondary and tertiary prevention, especially because mind body wellness approaches are not sufficiently reimbursed by 3rd party and governmental payers. With this in mind online web-based programs and cell phone applications will be of key importance in mind body public health and clinical efforts. Furthermore a segment of the public that is difficult to reach with face-to-face and with web-based programming–namely the socially phobic, those with post-traumatic stress disorder, autistic spectrum populations, and those with any illness that limits mobility will benefit from the use of virtual world mind body programming.

Future research in this area faces several interesting challenges. The technology is rapidly evolving. SL, in particular, has changed significantly since the completion of this study. The company has reduced some services, eliminated nonprofit/educational pricing and made little effort to improve user security. Additionally, the client-server approach employed by Linden Lab is being supplanted by distributed virtual environments like jibe developed by ReactionGrid. The interface to these virtual worlds is changing rapidly, becoming more intuitive but also more powerful. The rapid pace of these changes means that clinical programs developed using this technology may be obsolete by the time a comparative effectiveness trial can be completed.

The rapidly changing technological landscape also makes the choice of an appropriate comparison group difficult. A reasonable approach would be to compare a virtual intervention for stress reduction to the standard of care, which is a face-to-face intervention. However, since the human-computer interface is so critical to the intervention, an additional comparison of experienced computer users to those with little online experience may also be warranted.

Research into the provision of health interventions in virtual environments should continue in spite of these hurdles. The population is living longer with a variety of chronic diseases that can limit mobility. More and more healthcare will take place at the patient's home and on line. Fortunately, even the aging population is becoming increasingly comfortable with on line commerce, social network and and personal interactions.
