# Correction: The Feasibility and Impact of Delivering a Mind-Body Intervention in a Virtual World

**DOI:** 10.1371/journal.pone.0172863

**Published:** 2017-02-21

**Authors:** Daniel B. Hoch, Alice J. Watson, Deborah A. Linton, Heather E. Bello, Marco Senelly, Mariola T. Milik, Margaret A. Baim, Kamal Jethwani, Gregory L. Fricchione, Herbert Benson, Joseph C. Kvedar

There are errors in the Competing Interests section. The complete Competing Interests statement is: DAL served as the administrative assistant for the Partners Information Services Research Council at the same time that she was an active member of the research team. This does not alter the authors' adherence to all the PLoS ONE policies on sharing data and materials. The following authors hold or have held positions at the Benson-Henry Institute for Mind Body Medicine at Massachusetts General Hospital, which is paid by patients and their insurers for running the SMART-3RP and related relaxation/mindfulness clinical programs, markets related products such as books, DVDs, CDs and the like, and holds a patent pending (PCT/US2012/049539 filed August 3, 2012) entitled "Quantitative Genomics of the Relaxation Response": DBH, MTM, MAB, GLF, HB.
